# Meeting Postpartum Women’s Family Planning Needs Through Integrated Family Planning and Immunization Services: Results of a Cluster-Randomized Controlled Trial in Rwanda

**DOI:** 10.9745/GHSP-D-15-00291

**Published:** 2016-03-25

**Authors:** Lisa S Dulli, Marga Eichleay, Kate Rademacher, Steve Sortijas, Théophile Nsengiyumva

**Affiliations:** a FHI 360, Durham, NC, USA; b Institute for Reproductive Health, Kigali, Rwanda

## Abstract

**Objective:**

The primary objective of this study was to test the effectiveness of integrating family planning service components into infant immunization services to increase modern contraceptive method use among postpartum women.

**Methods:**

The study was a separate sample, parallel, cluster-randomized controlled trial. Fourteen randomly selected primary health facilities were equally allocated to intervention (integrated family planning and immunization services at the same time and location) and control groups (standard immunization services only). At baseline (May–June 2010), we interviewed postpartum women attending immunization services for their infant aged 6 to 12 months using a structured questionnaire. A separate sample of postpartum women was interviewed 16 months later after implementation of the experimental health service intervention. We used linear mixed regression models to test the study hypothesis that postpartum women attending immunization services for their infants aged 6–12 months in the intervention facilities will be more likely to use a modern contraceptive method than postpartum women attending immunization services for their infants aged 6–12 months in control group facilities.

**Results:**

We interviewed and analyzed data for 825 women from the intervention group and 829 women from the control group. Results showed the intervention had a statistically significant, positive effect on modern contraceptive method use among intervention group participants compared with control group participants (regression coefficient, 0.15; 90% confidence interval [CI], 0.04 to 0.26). Although we conducted a 1-sided significance test, this effect was also significant at the 2-sided test with alpha = .05. Among those women who did not initiate a contraceptive method, awaiting the return of menses was the most common reason cited for non-use of a method. Women in both study groups overwhelmingly supported the concept of integrating family planning service components into infant immunization services (97.9% in each group), and service data collected during the intervention period did not indicate that the intervention had any negative effect on infant immunization service uptake.

**Conclusion:**

Integrating family planning service components into infant immunization services can be an acceptable and effective strategy to increase contraceptive use among postpartum women. Additional research is needed to examine the extent to which this integration strategy can be replicated in other health care settings. Future research should also explore persistent misconceptions regarding the relationship between return of menses and return to fertility during the postpartum period.

## Abstract (precis)

Integrating contraceptive services into infant immunization services was effective, acceptable, and feasible without negatively affecting immunization uptake. Yet unmet need for contraception remained high, including among a substantial number of women who were waiting for menses to return even though, at 6 months or more postpartum, they were at risk of an unintended pregnancy. More effort is needed to educate women about postpartum return to fertility and to encourage those desiring to space or limit pregnancy to use effective contraception.

## INTRODUCTION

Healthy timing and spacing of pregnancies (HTSP) improves the health of both mothers and their children.^1^^-^^9^ Risks of miscarriage, abortion, and maternal death are much greater when births are spaced less than 2 years apart.^2^^-^^5^^,^^8^ Preterm birth, low birth weight, stillbirth, and newborn death are also more likely when births are spaced too closely together.^2^^,^^7^^-^^10^

Unmet contraceptive need is high for many postpartum women in sub-Saharan Africa; across 21 low- and middle-income countries, an estimated 61% of postpartum women had unmet contraceptive need.^11^ The extended postpartum period, 12 months after childbirth, can be a time of particularly elevated risk for an unplanned pregnancy. Research indicates as many as 40% of women who state they intend to use a contraceptive method 0–12 months postpartum do not do so.^12^

Many postpartum women have unmet need for contraception.

To reduce unmet contraceptive need, postpartum women need access to family planning information and services, yet reaching these women in Africa is often difficult because many do not deliver within health facilities and even fewer attend routine postpartum visits. Most postpartum women do, however, seek routine health services for their infants, including for immunizations. Given their timing, infant immunization services provide an important opportunity to reach postpartum women repeatedly throughout the postpartum period.

Infant immunization services can be an effective contact point for reaching postpartum women with family planning services.

Health service integration has become an important topic of discussion in global health. Intuitively, moving from vertically administered services to an integrated platform has the potential to improve service delivery efficiency, access, and uptake. Infant immunization services, with their worldwide success, have been an important focus for many integration efforts.^14^ A variety of maternal and child health services has been integrated into immunization services, including vitamin A supplementation, deworming, malaria prevention, nutrition, and HIV services, and there have been some efforts to integrate family planning with infant immunization.^14^

Although integrated family planning and infant immunization services are not new, limited evidence exists to support its effectiveness. One study in Togo (1994) demonstrated that a simple family planning referral message delivered during immunization services increased the number of family planning clients by over 50% without decreasing immunization service use.^15^ A second study in rural Bangladesh (2001) showed that introducing integrated family planning and child immunization services increased contraceptive prevalence from 28% to 53%.^16^ However, a more recent (2009–2010) cluster-randomized controlled trial in Ghana and Zambia that used screening and referral to family planning services demonstrated no increase in contraceptive method use among postpartum women attending infant immunization.^17^ The remaining evidence to support the strategy is largely derived from observational studies or programmatic experiences.^18^^,^^19^

The situation in Rwanda reflects that of many other countries in the region. Despite improvements in health care access and use in the past decade, facility-based deliveries and postpartum care remain underutilized. Nearly one-third of women in Rwanda deliver their babies at home, and only one-fifth receive any postpartum care.^20^ However, Rwanda has one of the most successful infant immunization programs in Africa.^21^ According to 2010 Demographic and Health Survey estimates, 86% of children under 2 in Rwanda received all recommended vaccinations within their first year.^20^

With high immunization attendance levels, immunization services could be an effective contact point for reaching postpartum women with family planning services in Rwanda. As such, we developed an intervention, using the Health Belief Model (HBM), to integrate elements of family planning services into infant immunization. The HBM model is one of the most widely used conceptual frameworks guiding the design of health behavior interventions and has been applied to sexual risk behaviors and contraceptive behaviors in a variety of populations including populations in sub-Saharan Africa.^22^ Given the dearth of existing evidence, this study was designed to test the effectiveness of this enhanced offering of family planning services during infant immunization visits to increase contraceptive method use among postpartum women.

## METHODS

### Study Design

The study was a separate sample, parallel, cluster-randomized controlled trial of a health services intervention designed to improve family planning use, thus reducing unmet contraceptive need among postpartum women attending public health care facilities in Rwanda. A cluster-randomized design was selected due to the design of the experimental intervention, which was delivered to both individuals and in group educational settings. Fourteen public primary health care facilities were randomly selected from a national sampling frame then randomly allocated to intervention or control groups of equal size (7 intervention facilities and 7 control facilities). A structured questionnaire was administered to postpartum women attending immunization services for their infant ages 6 to 12 months in all 14 study facilities during the baseline period (May–June 2010), immediately followed by intervention implementation in intervention group facilities (beginning in July 2010).

After baseline data were collected, intervention group immunization and family planning providers attended a 3-day training on postpartum family planning and the use of a screening tool to assess pregnancy risk among postpartum women. Providers in the control facilities received no training and continued to deliver services as usual. The endline questionnaire was administered to a separate sample of postpartum women 16 months after the beginning of intervention implementation.

Cost data were extracted from financial reports and confirmed with project personnel. To ensure intervention implementation fidelity, a study staff and a district Ministry of Health officer carried out quarterly supportive supervision visits.

### Intervention Description

The primary goal of the intervention was to increase uptake of family planning methods among postpartum women, thus reducing unmet contraceptive need. This goal was to be accomplished through enhanced components of family planning services delivered during infant immunization services designed to improve access to family planning services and to improve knowledge with regard to postpartum family planning. The intervention included 4 distinct yet interrelated components that were delivered to women attending all infant immunization services (i.e., at 6, 10, and 14 weeks and 9 months post-delivery).

1. **Concise messages delivered during group education sessions.** Immunization service providers routinely provide education to women attending immunization services on a variety of health topics. The delivery of such information did not change in intervention facilities, but the content on family planning was strengthened and delivered at each session, in addition to any other information being delivered. In intervention facilities, providers included the following information in all pre-immunization group education talks in the facility:
  - The risk of becoming pregnant after delivery of a baby if a woman is sexually active and not using a modern contraceptive method (which included the Lactational Amenorrhea Method [LAM] for women less than 6 months postpartum), even if she is breastfeeding or if her menses had not returned
  - The benefits of family planning to help women and their families time and adequately space their pregnancies for the health of the mother and her baby
  - Safe and effective contraceptive options for women to use during the postpartum period, even if breastfeeding
2. **A simple brochure in the local language (Kinyarwanda) distributed during group education.** The brochure contained messages about LAM, return to fertility and pregnancy risk during the postpartum period, the benefits of spacing pregnancies by at least 2 years, and contraceptive options for postpartum women. Brochures (Supplementary Material 1) were provided to women so they could share the information with their husbands.
3. **Individual screening of all women attending infant immunization services by the immunization provider.** An immunization provider met one-on-one with each mother, during which the provider asked the mother about the 3 LAM criteria to screen her for current risk of becoming pregnant. The provider also offered a brief counseling message depending upon risk classification and referral to family planning services for those currently or soon-to-be at risk of pregnancy. Providers were trained on a job aid (Supplementary Material 2) to assist the screening and counseling process. The screening questions were asked either as the baby received his/her immunization or by a separate provider while women were waiting for their baby’s immunization.
4. **Convenient offer of family planning services to women attending immunization at the same facility and on the same day as immunization services**. A family planning provider was available to receive clients as they were referred from immunization for family planning information/counseling and to initiate the woman’s method of choice, per the facility’s family planning service standard. No changes were made to the way in which family planning services were delivered with the exception of timing of availability; however, family planning service providers did receive reinforcement on the safety and appropriate timing of modern contraceptive methods for use by postpartum women for both those who did breastfeed and for those who did not.

**Figure:**
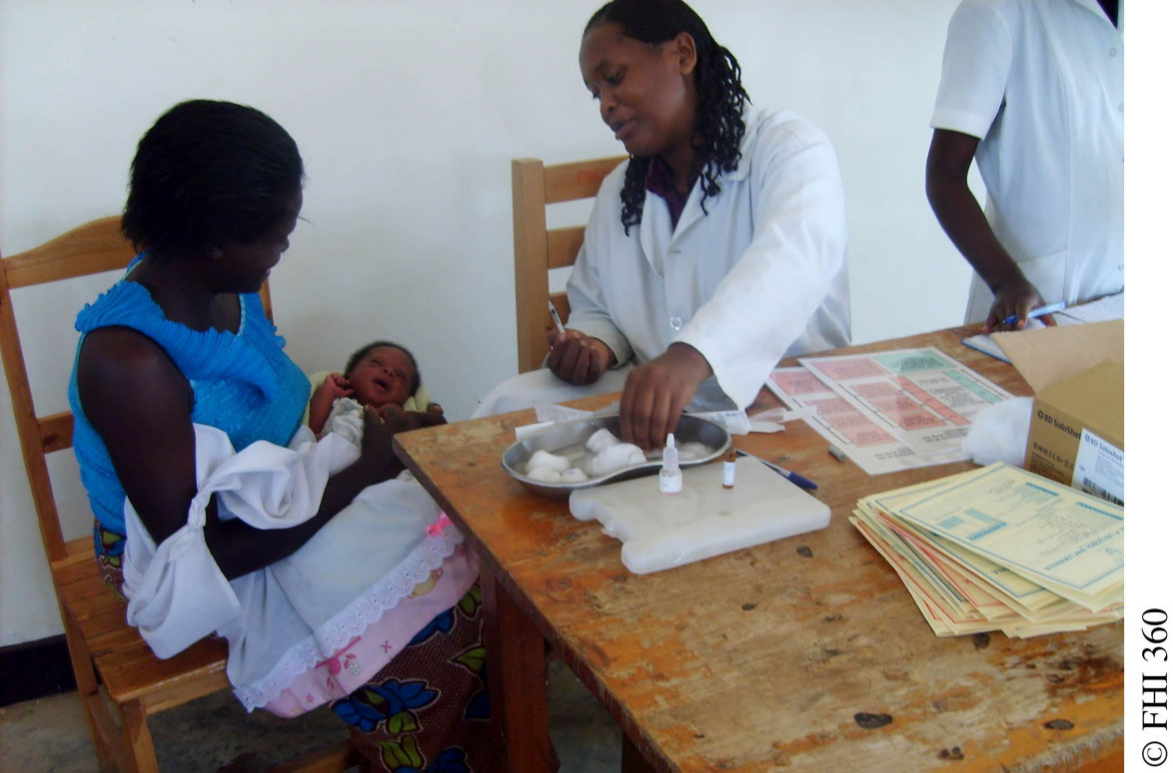
A woman attending infant immunization services in Rwanda also receives screening for family planning services.

**FIGURE 1.**
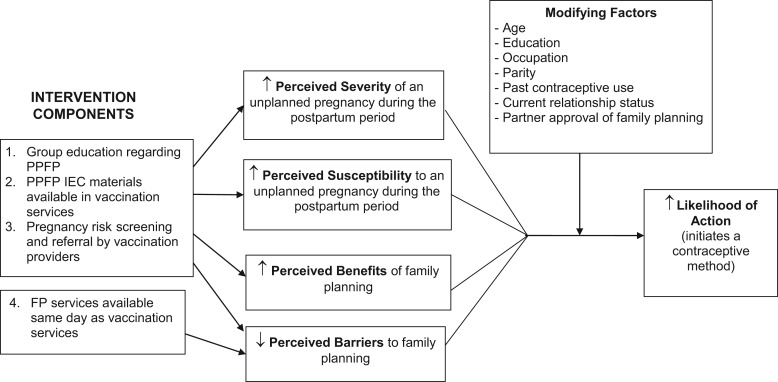
Conceptual Framework of Anticipated Intervention Effects Based on the Health Belief Model Abbreviations: FP, family planning; IEC, information, education, and communication; PPFP, postpartum family planning.

The brochure and key messages delivered through group education were adapted from the Extending Health Service Delivery (ESD) project and the Access to Clinical and Community Maternal, Neonatal and Women's Health Services (ACCESS-FP) project, both funded by the United States Agency for International Development (USAID).^23^^-^^25^ In addition to improving basic knowledge about postpartum family planning, the materials were also informed by the Health Belief Model. Specifically, the intervention was designed to act on women’s perceptions, including perceived severity of an unplanned pregnancy, perceived susceptibility to an unplanned pregnancy if sexually active and not using a contraceptive method, perceived benefits of contraception to prevent an unplanned pregnancy, and perceived barriers to accessing family planning services (Figure 1).

**Figure:**
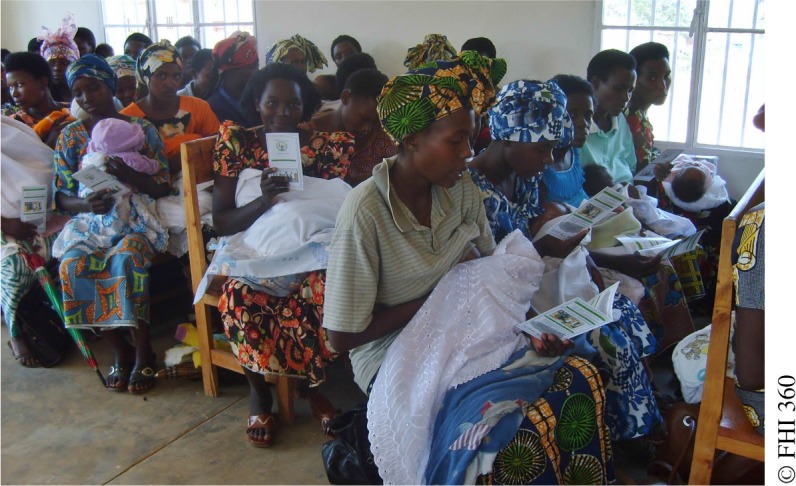
In Rwanda, clients review the integration project's brochure about family planning while awaiting immunization for their infants.

To accommodate the differing staffing and physical structure of health centers, each facility was asked to determine the most appropriate way to structure their integrated service delivery. We considered this ability to structure the service delivery according to the local context as an important element for sustainability. All facilities opted to offer family planning services concurrently with immunization so that once a woman was finished with her infant’s immunization she could see the family planning provider directly.

In addition to these 4 main intervention components, District Health Managers (DHMs), who already conducted routine supervision visits to health care facilities, were accompanied by a study staff member to observe the integrated service delivery on a quarterly basis. The DHM and study staff used a checklist designed by the study to assess the quality of the services being offered and to reinforce activities when necessary. This information was also used by the study to ensure the intervention was being delivered as intended and to take corrective action if necessary.

To put the intervention in place, immunization service providers, family planning services providers, and facility supervisors from intervention group facilities participated in a 3-day training session. This training covered essential postpartum family planning topics and the intervention components, in addition to a general refresher on family planning methods that the Ministry of Health opted to add. Training materials were also adapted from the ESD and ACCESS-FP materials. During the course of the study, it was observed that several trained providers had been transferred out of some of the intervention facilities leaving few or no providers who had undergone the initial training, so a 1-day refresher training on the intervention was carried out in each of the 7 intervention facilities. Health care providers and facilities in the control group received no additional training or support.

### Study Sample

The sample size of postpartum women was calculated to be able to detect a 12 percentage point difference in current modern contraceptive method use from baseline to endline between the intervention and the control groups (difference of differences), assuming a baseline contraceptive prevalence of 27% based on DHS data available at the time.^26^ Our primary sampling unit (PSU) was public primary health care facilities, and our secondary sampling unit included postpartum women who brought their infants for immunization. Adjusting for clustering at the facility level and assuming an intra-class correlation of 0.023 based on similar work in Madagascar,^27^ we estimated that a sample of 55 women interviewed at each time point in each of 14 health facilities, for a total of 770 women (385 participants per study arm) at both baseline and endline, would have 80% power to detect an intervention effect for a 1-sided test at the .05 significance level.

**FIGURE 2.**
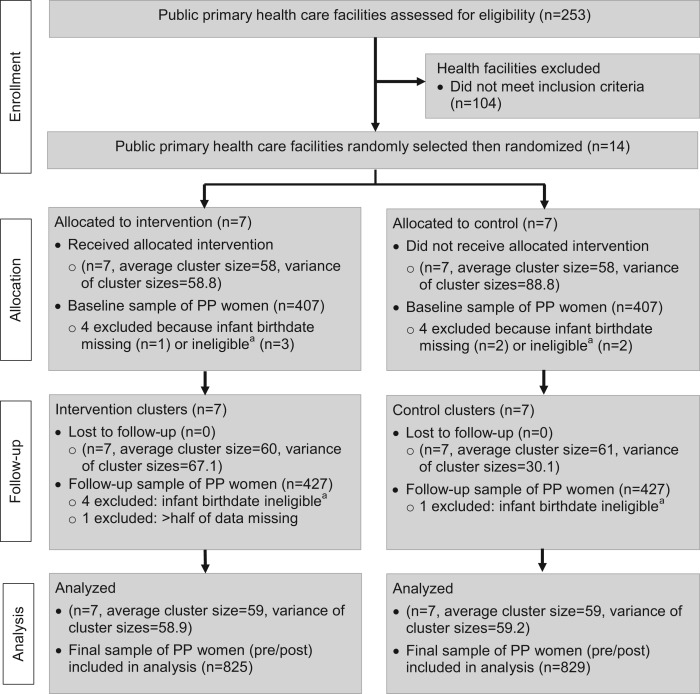
CONSORT Flow Diagram for Rwanda Postpartum Family Planning–Immunization Integration Study Abbreviation: PP, postpartum. ^a^ Women were recruited if their babies were between 6 months to 1 year old (180–365 days), as calculated by their birthdate. In a handful of cases, mothers of infants who were near but outside the range of 180–365 days completed interviews because they reported their infants as being of eligible age, but the age differed slightly once calculated in days and were thus excluded from analyses.

At the time of the study, there were 253 public primary health care centers in Rwanda (Figure 2). The sampling frame was restricted to public primary health care facilities with an average monthly client volume of at least 50 infant measles immunizations (n = 149) to ensure data collection could be completed within a reasonable time frame. Because health care is decentralized to the district level and district health managers supervise all facilities monthly, there was concern that if more than one facility was selected from a district and if the facilities were randomized to different treatment groups, there could be contamination across facilities. Therefore, we stratified the sampling frame by districts and then selected a random sample such that no more than one facility was sampled in any district, using the “Surveyselect” procedure in SAS System for Windows, version 9.3.^28^ Random allocation of facilities to treatment arms was also carried out using SAS/STAT software, version 9.3.^28^

Within the health centers, women were enrolled to participate in the study in the order they arrived during data collection until the sample size was achieved in each facility. Eligible participants included adult women, 21 years or older, or women 18 to 20 who had achieved legal majority status by emancipation due to marriage, who brought their own infants between 6 and 12 months of age to immunization services. We sampled women who were 6 to 12 months postpartum so that all women in the sample who desired to initiate a modern contraceptive method to delay or limit a new pregnancy should have done so by that point, if they followed the information and counseling being offered through the intervention. Prior to 6 months, some women may have been actively, or passively, protected by LAM; however, the intervention messaging clearly indicated to women that by 6 months postpartum, they should initiate a contraceptive method besides LAM if they wished to use family planning.

### Data Collection and Measures

Trained data collectors obtained written informed consent from all study participants and then interviewed study participants using structured questionnaires during exit interviews conducted in a private setting within the health facility. Data were collected on: (1) participant demographics; (2) reproductive and family planning history; (3) current pregnancy risk (infant age, breastfeeding status, status of menses, return to sexual activity); (4) postpartum family planning knowledge; and (5) perceptions toward postpartum family planning, including perceived susceptibility to and severity of an unplanned pregnancy, perceived benefits of family planning, perceived barriers to using family planning, and self-efficacy for using family planning. Participants in both groups were asked about the acceptability of integrating family planning services into infant immunization services and on satisfaction with their immunization service visit that day.

The primary outcome variable was self-reported current modern contraceptive method use. Modern methods comprised oral contraceptive pills, injectable contraceptives, contraceptive implants, female or male sterilization, intrauterine devices, male or female condoms, spermicides, or the Standard Days Method. For this study, emergency contraception was not counted as a modern method. LAM was not included because all participants were more than 6 months postpartum. Because population-level data demonstrate that family planning use increases with woman’s age, parity, and education and is associated with relationship status and partner approval, we included these as control variables.^20^^,^^26^ Occupation, which is related to education, was also included as a control variable.

Immunization service data, including the monthly number of immunization clients by immunization type (e.g., measles, polio, etc.), were collected for the duration of the intervention beginning July 2010. Data on costs associated with carrying out the intervention were also recorded, including material development and reproduction, staff training, and quarterly supportive supervisory visits.

### Data Analysis and Hypotheses

The main hypothesis tested in this study was that postpartum women who attend immunization clinic services for their infants ages 6–12 months in the intervention facilities will be more likely to use a modern contraceptive method than postpartum women who attend immunization clinic services for their infants, ages 6–12 months, in control group facilities. We tested this hypothesis with a linear mixed regression model using a 1-sided test at the .05 significance level with 12 degrees of freedom. The model controlled for age, parity, education, occupation, current relationship status, and perceived partner family planning approval, and it accounted for clustering by facility and time.

We also used linear mixed regression models to examine differences between groups and time for all descriptive variables separately and for bivariate analyses between individual characteristics and current modern contraceptive method use. The same approach was used to test differences between study groups and HBM variables. All models accounted for cluster effects. Analyses were carried out with the SAS System for Windows, version 9.3.^28^

Costing analyses were carried out using Microsoft Excel. We calculated the cost of the intervention per facility and estimated both the cost per full exposure, assuming 4 immunization visits over the 12-month extended postpartum period, and cost of the intervention per new acceptor. We used the number of babies who received measles vaccines from January–September 2011 (N = 5,036) in the intervention facilities as a proxy for the number of women who received the full 4-visit exposure. Because this was a 2-group, separate sample study, we did not have the actual number of new family planning acceptors. Instead, the number of family planning acceptors was calculated based on the number of women estimated to have been exposed to the intervention multiplied by the effect of the intervention shown in the study. We then divided the total incremental cost of the intervention by that number.

### Ethics Approval

This study was approved by the Rwanda National Ethics Committee and FHI 360's Protection of Human Subjects Committee, and it is registered on the US National Institutes of Health ClinicalTrials.gov database, registry #NCT01115361.

## RESULTS

### Participant Characteristics

The final sample included in the analysis comprised 825 women from the intervention group and 829 women from the control group. Study participants were demographically similar in intervention and control groups at baseline and endline (Table 1). Average age among participants was 27–28 years, and nearly all were Christian—fairly evenly divided between Protestantism and Catholicism. Most women had at least a primary school education, and more than three-fourths were literate. Just over half of all women reported working outside the home.

**TABLE 1:** Background Characteristics of Study Participants by Intervention Group and Time

Characteristic	Baseline		Endline	
	Control (n = 403)	Intervention (n = 403)	Control (n = 422)	Intervention (n = 426)
Age of mother, years, mean	27.9	28.4	27.4	28.5
No. of months postpartum, mean	9.4	9.4	9.4	9.4
Married/living with partner, %	96.5	97.5^†^	93.8	93.4^†^
Currently works outside the home, %	64.3	68.7	60.0	51.6
Literate, %	77.2	80.0	78.4	79.8
Education, %				
None	14.9	12.9	18.5	14.8
Primary	69.2	74.0	67.5	73.7
Secondary or more	15.9	12.7	14.0	11.5
Religion, %				
Catholic	40.7	49.1	35.8	46.5
Protestant	33.0	35.0	41.7	39.0
7th Day Adventist	15.6	10.7	15.6	8.9
Muslim	5.7	2.0	1.4	2.1
Other	4.0	2.7	5.4	3.5
No. of living children, mean	2.5	2.6	2.4	2.7

† Statistically significant difference between baseline and endline within group at *P*=.05.

### Contraceptive Use and Unmet Need

Modern contraceptive method use was relatively high among study participants; roughly half of women across study groups at both time points were using a modern contraceptive method (Table 2). Unmet contraceptive need was also high (45.6% in the control group and 39.2% in the intervention group at endline); nearly all women not currently using a modern method desired to space or limit their births (Table 2). Just under half of respondents reported desiring no more children; among those who desired additional children, nearly all wanted to wait at least 2 years before their next pregnancy. Most respondents in both groups at both time points had returned to sexual activity since childbirth.

Modern contraceptive use was relatively high among all study participants, but unmet need was also high.

**TABLE 2:** Modern Contraceptive Use and Unmet Need for Contraception by Intervention Group and Time

	Baseline		Endline	
	Control (n = 403)	Intervention (n = 403)	Control (n = 422)	Intervention (n = 426)
Currently using a modern method, %	57.5	49.0†	50.7¥	57.0^†^^¥^
Injectables	38.6	28.4	29.2	31.0
Pills	8.2	12.4	6.9	10.6
Male condoms	5.0	3.4	7.1	7.5
Implants	3.2	2.5	4.7	4.2
Standard Days Method	1.5	1.7	1.0	1.0
IUD	0.5	0.0	1.0	1.1
Female sterilization	0.3	0.5	0.5	0.2
Male sterilization	0.3	0.0	0.0	0.0
Female condoms	0.0	0.0	0.0	0.5
Sexually active, %	92.8	91.5	94.1	92.7
Desires pregnancy in <2 years, %	4.3	2.6	4.0	3.8
Unmet need for contraception, %	38.2	48.4	45.3	39.2
Unmet need to space	16.4	24.8	19.9	19.2
Unmet need to limit	21.8	23.6	25.4	20.0

† Statistically significant difference between baseline and endline within group at *P* = .05.

¥ Statistically significant difference between groups at *P* = .05.

### Intervention Effect

To assess the effectiveness of the intervention to increase modern contraceptive method use, we examined the change in modern method use between intervention and control groups across time points (Table 3). Results showed that the intervention had a statistically significant and positive effect on modern method use among intervention group participants compared with control group participants (regression coefficient, 0.15; 90% confidence interval [CI], 0.04 to 0.26). In other words, we observed an 8% increase in the intervention group and a 7% decrease in the control, resulting in a 15 percentage point difference between the intervention and control groups when comparing baseline to follow-up results. Although we conducted a 1-sided significance test, this effect was also significant at the 2-sided test with an alpha = .05 (95% CI, 0.01 to 0.29).

The integrated intervention had a significant and positive effect on modern method use.

**TABLE 3:** Three Linear Mixed Models of Modern Contraceptive Use Over Time, for Intervention Group Alone, Control Group Alone, and Comparing Intervention With Control Group

(Outcome is current method use)	Regression Estimate	95% CI	90% CI
Intervention only (Endline–Baseline)	0.09†	-0.004, 0.19	0.01, 0.17
Control only (Endline–Baseline)	-0.01	-0.16, 0.04	-0.14, 0.02
Difference of differences	0.15††	0.01, 0.29	0.04, 0.26

† *P* = .10;

†† *P* = .05.

Model adjusted for mother’s age, parity, marital status, education, and religion and accounts for clustering by facility and facility*time.

### Health Belief Model Effects

Concerning the HBM concepts and contraceptive method use, women with a higher perceived susceptibility to an unplanned pregnancy were more likely than those with a lower perceived susceptibility to use a modern contraceptive method at both baseline and follow-up (linear mixed model regression estimate, 0.24; *P* = .05) (Table 4). Greater perceived severity of an unplanned pregnancy and perceived benefits of family planning were also significantly and positively associated with greater family planning use (regression estimates, 0.04 and 0.06, respectively). Greater perceived barriers to family planning use was associated with lower family planning use (regression estimate, -0.14); however, this finding was not statistically significant.

**TABLE 4:** Bivariate Analyses of Health Belief Model (HBM) Concepts and Contraceptive Method Use Using Linear Mixed Models

HBM Concept	Regression Estimate
Perceived susceptibility to unplanned pregnancy	0.24†
Perceived severity of unplanned pregnancy	0.04†
Perceived benefits of FP	0.06†
Perceived barriers to receiving FP service	-0.14

† *P* = .05.

Model accounts for clustering by facility and facility*time.

We found a small but statistically significant change in perceived susceptibility to an unplanned pregnancy between intervention and control groups from baseline to follow-up; perceived susceptibility increased from baseline to endline in intervention facilities and decreased in control facilities. However, no other significant changes were observed among HBM concepts because perceptions of severity of an unplanned pregnancy and benefits of family planning were already very high in both groups, and perceived barriers to family planning were relatively low (data not shown).

### Reasons for Non-Use

At endline, we asked those not currently using a modern method (non-users) their reasons for non-use (data not shown). Responses were similar in both groups; the most common reason reported was that participants were awaiting menses to return to initiate a method (46.1% in the control group, 50.3% in the intervention group). Other important reasons for not using a contraceptive method included fear of side effects or health problems associated with family planning (19.4% in the control group, 13.4% in the intervention group), as well as currently breastfeeding (8.2% in the control group, 11.2% in the intervention group).

Most non-users said they were waiting for menses to return before initiating a method.

### Postpartum Women’s Perspectives on Family Planning and Immunization Service Integration

At endline, women in both study groups were asked about their perspectives on integrating family planning services components into infant immunization services (Table 5). Women in both groups nearly universally (98%) agreed that infant immunization services were a good time to receive information on family planning options. Approximately three-quarters of all women also stated that they preferred to get family planning services on the same day when they bring their infants for immunizations. Fewer than 20% overall stated they did not think the immunization service visit was the appropriate time to receive family planning information; most of these women stated they preferred to come when they did not have their babies with them (data not shown).

**TABLE 5:** Postpartum Women’s Perspectives on Integration of Family Planning and Immunization Services and Satisfaction With Immunization Services at Endline, % Who Agreed With Statements

	Intervention (n = 427)	Control (n = 426)
Perspectives on integration		
It is good to get information about family planning options when I bring my baby for immunization.	97.9	97.9
I prefer to get both baby immunization and family planning on the same day rather than to come to the health facility on different days.	73.3	75.4
If my husband knew I received family planning information during immunization service, he would be unhappy.	9.1	10.8
Satisfaction with immunization services		
Immunization provider treated me respectfully.	98.6	98.1
Immunization provider gave me an opportunity to ask any health-related questions.	28.6	16.7
Immunization provider helped me get needed health information.	36.3	22.5
Wait time to see the provider who provided my child’s immunization was acceptable.	85.7	86.9
Overall satisfaction with the service received during visit		
Not satisfied at all	1.2	2.1
Somewhat unsatisfied	2.8	1.4
Somewhat satisfied	21.8	18.8
Very satisfied	74.2	77.7

Women in both groups were very satisfied with the services they received (Table 5). There was no difference between study groups in overall satisfaction, satisfaction with the wait time, or in the proportion of women who stated that providers treated them with respect. However, more women in the intervention group reported that they were given the opportunity to ask health-related questions and that the provider was able to give them the information they needed.

### Immunization Service Statistics

**FIGURE 3:**
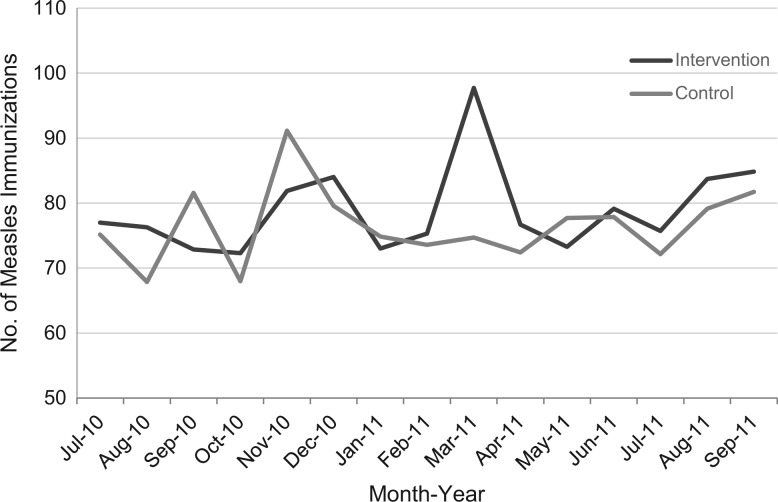
Average Number of Measles Immunizations per Month, by Study Group, During the Service Integration Intervention Period, From August 2010 to September 2011

Given the possibility that integrating family planning education, pregnancy risk screening, and family planning services could have a negative effect on immunization uptake, we collected service data on measles immunization (scheduled between 9 and 12 months of age) at all facilities (Figure 3). There was considerable monthly variation in the numbers of infants immunized at all facilities. Observed peaks are likely due to periodic community outreach efforts (such as Mother and Child Health Weeks, which occur in March and November each year) during immunization campaigns to reach unimmunized children. We observed no downward trend in the numbers of infants immunized for measles in the intervention facilities over the course of the study period, indicating that once family planning services were integrated into immunization services within intervention facilities, immunization uptake did not decrease. Although we present data only for the measles vaccine in Figure 3, trends were similar for all recorded vaccines, including the first 3 doses of diphtheria, tetanus, and pertussis vaccine (DTP).

There were no downward trends in the number of infants immunized for measles in the integrated intervention facilities.

### Costs of the Intervention

Intervention costs included:

- Document development and reproduction (job aid and brochures)
- Preparation visits to the intervention sites
- Training providers and supervisors
- Launching activities in intervention sites
- Quarterly supervisions to the intervention sites during implementation
- One-day refresher training for all intervention sites

After subtracting costs not relevant to scale-up (e.g., staff time to develop training materials), the total cost was US$24,203. This resulted in a cost of US$4.81 per woman for full exposure to the intervention (at 6, 10, and 14-week and at 6-month immunization visits). Assuming that 15% of women would accept a new contraceptive method, based on the 15 percentage point difference observed between the intervention and control groups, the intervention cost was US$32.06 per new family planning acceptor.

## DISCUSSION

This study contributes to the small global body of evidence on the effectiveness of integrated health service delivery and supports previous findings that integrating family planning service components into infant immunization services can be an effective, acceptable, and feasible strategy for increasing family planning service uptake among postpartum women. Clients were overall very positive about receiving family planning information and services during infant immunization, and integration did not adversely affect reported service satisfaction. We also demonstrated that adding family planning service components did not negatively affect immunization service uptake, which has been a concern among some public health professionals.^13^^,^^29^

Integrating family planning services into immunization programs can be effective, acceptable, and feasible.

The intervention sought not only to increase modern contraceptive method uptake but also to improve postpartum family planning knowledge among women. Given that half of non-users at endline stated that they were awaiting menses to return to initiate a method despite being 6 or more months postpartum, it appears that the intervention was not completely successful at dispelling this misconception. Our findings are consistent with several other investigations that have explored the relationship between postpartum amenorrhea and initiation of a family planning method, which found that many women await the return of menses before initiating a family planning method, potentially placing them at risk for an unintended pregnancy.^30^^-^^32^ More efforts are needed to ensure women and their providers understand that postpartum women can become pregnant before their menses return, even while breastfeeding, and that sexually active women who desire to space or limit their pregnancies should initiate an effective modern contraceptive method as early as possible. Additional research is needed to better understand the persistence of these misperceptions and to test strategies to address them.

More efforts are needed to ensure women understand that they can become pregnant before their menses return in the postpartum period.

The integration approach employed in this study permitted a degree of flexibility within health facilities with regard to how the family planning and immunization services were co-delivered, which we believe is important to successful implementation and sustainability. However, several systemic challenges affected integrated service implementation. We selected public primary health centers where both immunization and family planning services are offered. In Rwanda, family planning services are offered every day of the week, although not necessarily at the same time as immunization services. In settings where family planning services are not offered daily, this intervention may require significant changes to service delivery, which may or may not be feasible. We also found that a single training session for providers was insufficient to successful implementation and that supportive supervision was key to successful, ongoing implementation. Until providers are experienced in the new service delivery strategy, maintaining the level of supportive supervision provided in the study intervention could prove a challenge to some district health personnel.

We also found that, despite a conducive service delivery setting, provider attrition through transfers caused trained providers to be replaced with untrained providers, affecting service delivery. The most permanent comprehensive solution to this problem would be to include training on integrated family planning and immunization service delivery in preservice education for all providers and to systematically train all current primary care providers on the strategy through new or existing training opportunities.

At US$32.06 per new acceptor, this strategy could be considered relatively expensive; however, many expenses can be reduced or eliminated by incorporating the work into existing activities. Two of the largest expenses were provider training and quarterly supervision visits, both of which could be integrated into existing training and supervision activities.

### Limitations

This study used a rigorous study design; however, it is not without limitations. We used a separate sample approach to measure intervention effects. The primary reason for enrolling separate samples of postpartum women was to enroll women who could be fully exposed to the intervention. Although most women in Rwanda bring their babies to health facilities to receive immunizations, only about half of women delivered in health facilities at the time of the study.^26^ Sampling women at the time of delivery and following them during the postpartum period would have limited our sampling frame to only women who delivered in a facility as opposed to all women who bring their infants to immunization services. We believe the information gained through this sampling strategy outweighs its limitations. A second limitation was the relatively small number of facilities included in the study sample. Having only 7 PSU per study arm decreased the likelihood that randomization led to completely comparable study groups. In response, we chose to examine the difference in modern contraceptive method use from baseline to endline between study groups (difference of differences), reducing the study’s power to detect statistically significant differences. Despite this limitation, we observed a statistically significant and positive intervention effect.

### Study Implications

This rigorous trial demonstrated that integrating family planning service components into infant immunization services can be an effective, acceptable, and feasible strategy for increasing modern contraceptive use among postpartum women without negatively affecting immunization services. The study addressed some of the limitations previously noted in the literature on this topic by including a control group, collecting extensive process data to understand implementation fidelity, and collecting costing data.

Further research is needed to test this approach in other settings. Given that this is one of only a handful of research studies directly examining the effectiveness of integrated family planning and infant immunization services and that the study enrolled a relatively small number of health care facilities within one country setting, attempting to replicate these results elsewhere is important. Additionally, a strong policy environment aiming to improve maternal and child health, extensive engagement of the Ministry of Health in the planning and implementation of the intervention, as well as intensive efforts to strengthen the country’s health system likely played important roles in the success of the intervention. Replicating this work in settings where political or health system support is not as strong may not generate the same results.

In March 2013, the Rwanda Ministry of Health held a national meeting to discuss results of this and other studies focused on improving family planning services and contraceptive uptake. Participants recommended national scale-up of the intervention and initiated discussions on changes to service delivery guidelines, supervision requirements, training curricula, and data collection systems to support scale-up. Recommendations were sent to the national Maternal and Child Health Technical Working Group for incorporation into Ministry of Health and partner organization work plans.

**Figure:**
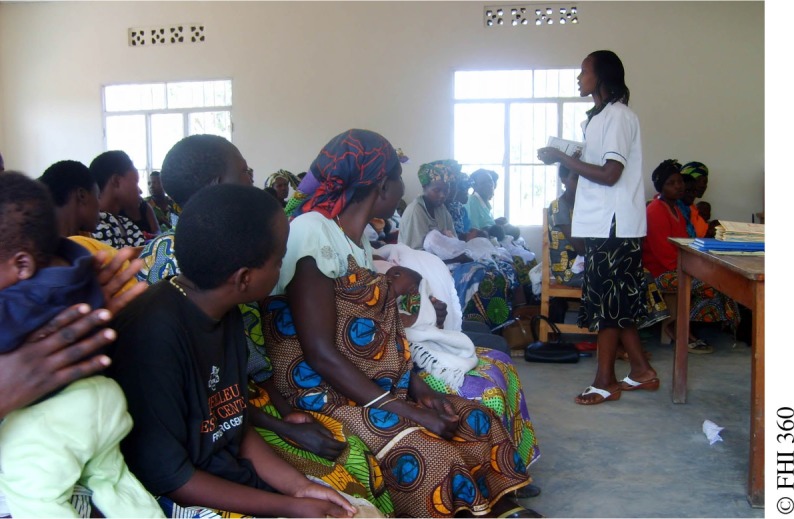
In Rwanda, a nurse provides information on postpartum family planning during a group education session while the women wait for immunization services.

## Supplementary Material

Supplementary Material 1

## Notes

**Peer Reviewed**

**First Published Online:** 2016 Feb 22
