# Supplementary material for: Meeting Postpartum Women’s Family Planning Needs Through Integrated Family Planning and Immunization Services: Results of a Cluster-Randomized Controlled Trial in Rwanda
Source: Glob Health Sci Pract. 2016 Mar 25;4(1):73–86. doi: 10.9745/GHSP-D-15-00291 (PMC4807750; doi:10.9745/GHSP-D-15-00291)
Supplement: Supplementary Material 1 [file GHSP-D-15-00291_index.html]

Supplement to Meeting Postpartum Women’s Family Planning Needs through Integrated Family Planning and Immunization Services: Results of a Cluster-Randomized Controlled Trial in Rwanda | Global Health: Science and Practice

## GHSP-D-15-00291 Supplementary Material

Client brochure. doi: 10.9745/GHSP-D-15-00291

- Supplementary Material - Client brochure. doi: 10.9745/GHSP-D-15-00291
- Supplementary Material - Provider job aid. doi: 10.9745/GHSP-D-15-00291
