# Supplementary material for: Meeting Postpartum Women’s Family Planning Needs Through Integrated Family Planning and Immunization Services: Results of a Cluster-Randomized Controlled Trial in Rwanda
Source: Glob Health Sci Pract. 2016 Mar 25;4(1):73–86. doi: 10.9745/GHSP-D-15-00291 (PMC4807750; doi:10.9745/GHSP-D-15-00291)
Supplement: Supplementary Material 1 [file 15-00291-Client-brochure-on-Supplementary-Material-1-PPFP-Client-brochure-on.pdf]

Bumwe mu buryo buhita bukoreshwa ukimara kubyara abantu benshi batamenya ni uburyo bw'agapira ko mu mura

Uburyo bw'agapira ko mu mura ni iki ?

Agapira ko mu mura ni agapulasitike gato, kazengurutswe n'umuringa gashyirwa mu mura w'umugore kakabuza intanga ngabo guhura n'intanga ngore.

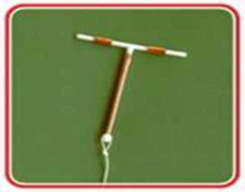

Ni nde ushobora gukoresha agapira ?

Umugore wese udatwite ashobora gukoresha agapira

Ni ryari ushobora gutangira gukoresha agapira nyuma yo kubyara ?

- Iyanyuma ikimara gusohoka, kugeza ku masaha 48 nyuma yo kubyara
- Uhereye kubyumweru 4 umaze kubyara na nyuma yaho

Ibyiza by'agapira

- Uburyo bwizewe
- Iyo ubufashe nta mvune yo guhora usubira kwa muganga.
- Uburyo bw'igihe kirekire.
- Imibonano mpuzabitsina ntihinduka
- Uburumbuke buhita bugaruka ukimara gukuramo agapira
- Uburyo bukoreshwa n'abagore bonsa
- Uburyo bw'ibanga

Ingaruka zishoboka ku mugore ukoresha agapira

- Ku bagore bake cyane bashobora kuribwa munda mugihe cy'imihango.
- Ku bagore bake cyane imihango ishobora kuba myinshi
- Ku bagore bake cyane agapira gashobora gusohoka

Wamenyesha nde ugize ikibazo cyangwa impungenge

Ugize ikibazo wamenyesha umuforomo cyangwa umuganga wese wo ku kigo nderabuzima cyangwa ibitaro bikwegereye.

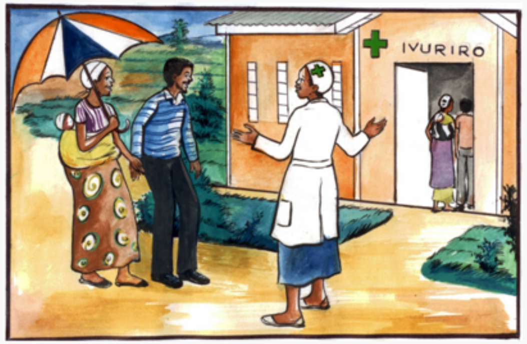

Ni he wabona agapira ? Ku kihe kiguzi?

- Agapira kaboneka mu bigo nderabuzima no mu bitaro bifite abakozi babihuguriwe
- Nk'ubundi buryo bwose bwo kuboneza urubyaro agapira nta kiguzi gasaba.

Gutegereza byibura imyaka 2 mbere yo kongera gusama bituma umubyeyi n'umwana bagira ubuzima bwiza, bikagabanya impfu z'abana n'ababyeyi

USAID FROM THE AMERICAN PEOPLE

fhi Family Health International

Jhpiego an affiliate of Johns Hopkins University

REPUBULIKA Y'U RWANDA

MINISITERI Y'UBUZIMA

KUBONEZA URUBYARO UKIMARA KUBYARA

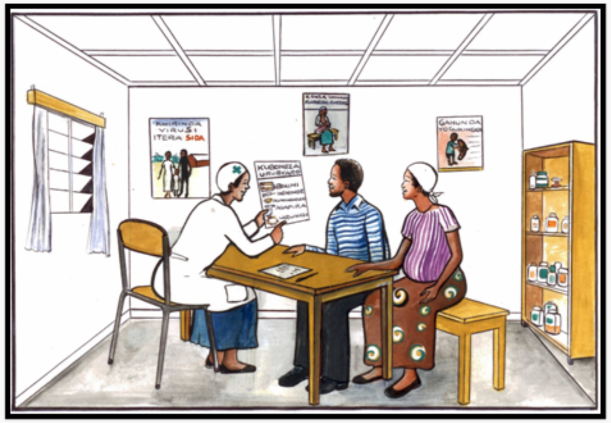

Umuryango wagombye gutekereza ku buryo bwo kuboneza urubyaro bagitwite, kubera ko umugore wese ashobora gusama adasubiye mu mihango nyuma yo kubyara iyo adakoresheje uburyo bwizewe bwo kuboneza urubyaro.

Uburyo bushoboka gukoreshwa nyuma yo kubyara

Hari uburyo bwinshi bwo kuboneza urubyaro bushobora gukoreshwa nyuma yo kubyara kugira ngo wirinde guhita wongera gusama

Akibyara kugeza ku masaha 48

- Uburyo bwo kwonsa ntakindi uha umwana
- Agapira ko mu mura
- Agakingirizo k'umugabo cyangwa umugore
- Ibinini bigizwe n'umusemburo umwe k'umugore utonsa
- Inshinge n'udupira two mu kuboko k'umugore utonsa.
- Gufunga burundu k'umugore
- Gufunga burundu k'umugabo

Kuva ku masaha 48 kugeza kubyumweru 6 nyuma yo kubyara

- Uburyo bwo kwonsa ntakindi uha umwana
- Agakingirizo k'umugabo cyangwa umugore
- Ibinini bigizwe n'umusemburo umwe k'umugore utonsa
- Inshinge n'udupira two mu kuboko ku mugore utonsa
- Ibinini by'imisemburo 2 ku mugore utonsa guhera ku byumweru bitatu
- Gufunga burundu k'umugabo

Kuva ku byumweru 6 kugeza ku mezi 6 nyuma yo kubyara

- Uburyo bwo kwonsa nta kindi uha umwana
- Agapira ko mu mura (gashobora gukoreshwa kuva ku byumeru 4)
- Ibinini bigizwe n'umusemburo umwe
- Inshinge n'udupira two mu kuboko
- Agakingirizo k'umugabo cyangwa umugore
- Ibinini bigizwe n'imisemburo 2 ku mugore utonsa
- Gufunga burundu k'umugore
- Gufunga burundu k'umugabo

Kuva ku mezi 6 kugeza ku mwaka nyuma yo kubyara na nyuma yaho

- Agapira ko mu mura
- Ibinini bigizwe n'umusemburo umwe
- Ibinini bigizwe n'imisemburo ibiri
- Inshinge n'udupira two mu kuboko
- Agakingirizo k'umugabo cyangwa umugore
- Gufunga burundu k'umugabo
- Gufunga burundu k'umugore
- Uburyo bw'urunigi iyo imihango yagarutse

Mubyeyi Igihe cyose ugiye kwa muganga nyuma yo kubyara (gukingiza umwana, kwisuzumisha nyuma yo kubyara, kwivuza, kuvuza umwana, ….); boneraho gusaba inama zo kuboneza urubyaro

Uburyo bukunze gokoreshwa

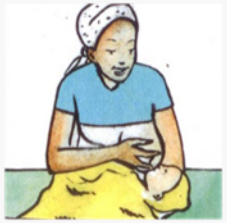

Konsa gusa

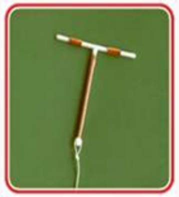

Agapira ko mumura

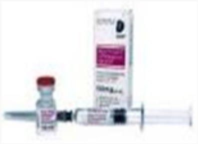

Urushinge

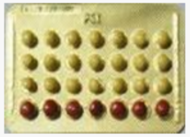

Ibinini

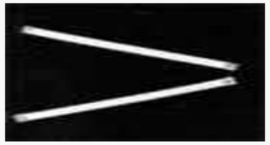

Udupira two mu kuboko

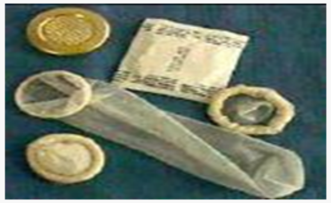

Agakingirizo k'abagabo

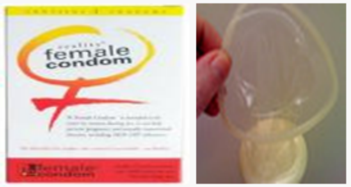

Agakingirizo k'Umugore

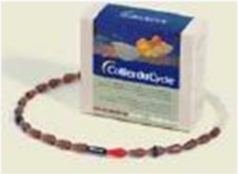

Urunigi

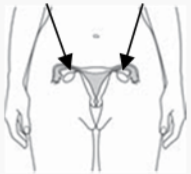

Gufunga k'umugore

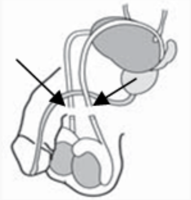

Gufunga k'umugabo
