# Supplementary material for: Meeting Postpartum Women’s Family Planning Needs Through Integrated Family Planning and Immunization Services: Results of a Cluster-Randomized Controlled Trial in Rwanda
Source: Glob Health Sci Pract. 2016 Mar 25;4(1):73–86. doi: 10.9745/GHSP-D-15-00291 (PMC4807750; doi:10.9745/GHSP-D-15-00291)
Supplement: Supplementary Material 1 [file 15-00291-Provider-job-aid-Supplementary-Material-2-PPFP-Provider-job-aid.pdf]

Front

Start here!

Determine A Mother's Need for Family Planning

No	Question	Yes	Result
No	Have your menses returned?	Yes	You are at risk for pregnancy now, even if you are fully breastfeeding.
No	Are you feeding your baby any other foods or liquids besides breastmilk?	Yes	You are at risk for pregnancy now, even though your menses have not returned.
No	Is your baby more than 6 months old?	Yes	You are at risk for pregnancy now even if you are fully breastfeeding and your menses have not returned.

- Your risk of pregnancy now is low— because you are naturally protected from pregnancy by a process known as the lactational Amenorrhea method (LAM).
- Your risk for pregnancy will increase soon. *See reverse*

- Women who delay another pregnancy for at least 2 years after giving birth are healthier and have healthier babies.
- To space pregnancies you need to use a FP method.

Refer mother to FP clinic

Back

Go for Family Planning when any ONE of these things occur:

1 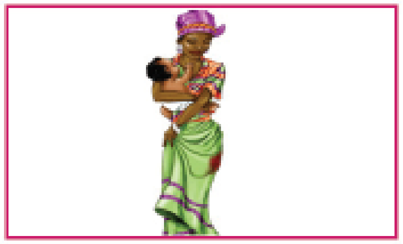 Your menstrual bleeding returns.

OR

2 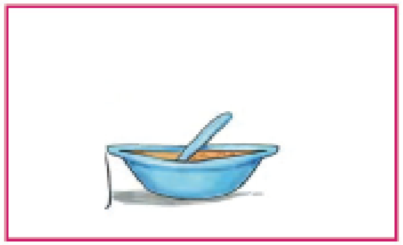 You feed your baby other liquids or foods besides breastmilk.

OR

3 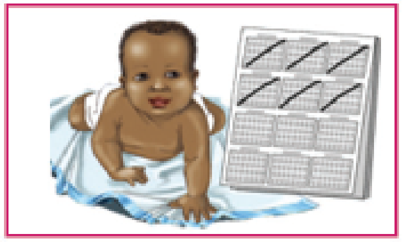 Your baby is 6 months old.

Why do you need another method?

When one of these conditions occurs, it is a sign that your fertility has returned. The LAM method will no longer be working.

To prevent having another baby too soon, you should use another family planning method.

Why is birth spacing important?

It is Healthy! Women who delay another pregnancy for at least 2 years after giving birth are healthier and have healthier babies.

USAID FROM THE AMERICAN PEOPLE · fhi360 THE SCIENCE OF IMPROVING LIVES · PROGRESS IN FAMILY PLANNING
